# Immunologic features of nontuberculous mycobacterial pulmonary disease based on spatially resolved whole transcriptomics

**DOI:** 10.1186/s12890-024-03207-2

**Published:** 2024-08-13

**Authors:** Jaemoon Koh, Sehui Kim, Joong-Yub Kim, Jae-Joon Yim, Nakwon Kwak

**Affiliations:** 1 Department of Pathology, Seoul National University Hospital, Seoul, South Korea; 2 Laboratory of Immune Regulation in Department of Biomedical Sciences, Seoul National University College of Medicine, Seoul, South Korea; 3 Department of Pathology, Korea University Guro Hospital, Seoul, Republic of Korea; 4 Division of Pulmonary and Critical Care Medicine, Department of Internal Medicine, Seoul National University College of Medicine, 101 Daehak-Ro, Jongno-Gu, Seoul, 110-744 South Korea

**Keywords:** Nontuberculous mycobacteria, Lung disease, Macrophage activation, M1 phenotype

## Abstract

**Background:**

The immunologic features of nontuberculous mycobacterial pulmonary disease (NTM-PD) are largely unclear. This study investigated the immunologic features of NTM-PD using digital spatial profiling techniques.

**Methods:**

Lung tissues obtained from six patients with NTM-PD between January 1, 2006, and December 31, 2020, at Seoul National University Hospital were subjected to RNA sequencing. Cores from the peribronchial areas were stained with CD3, CD68, and DNASyto13, and gene expression at the whole-transcriptome level was quantified using PCR amplification and Illumina sequencing. Lung tissues from six patients with bronchiectasis collected during the same period were used as controls. The RNA sequencing results were validated using immunohistochemistry (IHC) in another cohort (30 patients with NTM-PD and 15 patients with bronchiectasis).

**Results:**

NTM-PD exhibited distinct gene expression patterns in T cells and macrophages. Gene set enrichment analysis revealed that pathways related to antigen presentation and processing were upregulated in NTM-PD, particularly in macrophages. Macrophages were more prevalent and the expression of genes associated with the M1 phenotype (*CD40* and *CD80*) was significantly elevated. Although macrophages were activated in the NTM-PD group T cell activity was unaltered. Notably, expression of the costimulatory molecule *CD28* was decreased in NTM-PD. IHC analysis showed that T cells expressing Foxp3 or TIM-3, which facilitate the regulatory functions of T cells, were increased.

**Conclusions:**

NTM-PD exhibits distinct immunologic signatures characterized by the activation of macrophages without T cell activation.

**Supplementary Information:**

The online version contains supplementary material available at 10.1186/s12890-024-03207-2.

## Background

Nontuberculous mycobacteria (NTM), comprising more than 200 species other than *Mycobacterium tuberculosis* and *M. leprae*, occur ubiquitously in the environment, including soil, dust, and water [1]. NTM can cause chronic infections in humans [1] and have been associated with worse survival outcomes than tuberculosis (TB) infections [2]. Over the past few decades, the burden of NTM infections has rapidly increased. In South Korea, where the burden of TB is intermediate, the prevalence of NTM has increased from 11.4 to 56.7 cases per 100,000 people between 2010 and 2021, surpassing that of TB [3].

The primary defense against NTM infection is derived from Th1 immunity [4]. Upon inhalation of aerosolized NTM, the host initiates a macrophage-mediated immune response. Alveolar macrophages recognize specific molecular patterns, including mycobacterial lipoproteins or glycolipids, through Toll-like receptor (TLR) 2 and initiate the direct killing of mycobacteria and production of interleukin (IL)-12 [5]. IL-12 subsequently activates T cells to produce interferon (IFN)-γ, which further amplifies Th1 immunity. Consequently, defects in the IL-12/IFN-γ axis render individuals susceptible to NTM infections [6].

While the roles of the IL-12/IFN-γ axis in the pathogenesis of NTM infection have been extensively investigated, defects in these genes are very rare in patients with NTM-pulmonary disease (PD), accounting for 80–90% of NTM infections [7]. Defects in the IL-12/IFN-γ axis typically present as disseminated infections, whereas NTM-PD, a localized disease, likely involves different immune responses [5]. However, most studies have focused on immune responses derived from peripheral blood cells rather than lung tissues, thus failing to capture the immunologic signature inherent in NTM-PD.

NTM-PD is closely associated with bronchiectasis; preexisting bronchiectasis, characterized by airway dilatation leading to the impairment of mucociliary clearance and persistent airway inflammation, is an important predisposing factor [8]. Approximately 80% of patients with NTM-PD present with bronchiectasis at the time of diagnosis [9]. Given these shared characteristics, we hypothesized that identifying the immunologic features distinct from bronchiectasis could provide valuable insights into the immunologic landscape of NTM-PD.

In this study, we utilized the digital spatial profiling (DSP) technique, which enables quantitative profiling of gene expression in specific cells within spatially defined regions [10], to analyze the immunologic signatures of NTM-PD in lung tissues obtained from patients who underwent surgical resection for NTM-PD and non-NTM bronchiectasis (referred to as bronchiectasis hereafter).

## Methods

### Study population

The study included patients diagnosed with NTM-PD between January 1, 2006, and December 31, 2020, at Seoul National University Hospital (Seoul, South Korea). These patients underwent surgical resection as adjunctive therapy because of persistently positive mycobacterial cultures, long-standing cavities, radiographic aggravation, or massive hemoptysis [11]. Lung tissues obtained from six patients were subjected to DSP analysis, while tissues from an additional 30 patients were used for immunohistochemistry (IHC).

As controls, lung tissues were obtained from patients with bronchiectasis who underwent surgical resection as an adjunctive treatment during the same period (six for DSP analysis and 15 for IHC). The absence of NTM infection in non-NTM-PD-bronchiectasis was confirmed by negative mycobacterial cultures on at least two occasions at intervals of > 1 month.

This study was conducted in accordance with the Declaration of Helsinki. The Institutional Review Board of the Seoul National University Hospital approved the study protocol (IRB No. 2009-048-1155).

### Tissue microarray (TMA) construction

**Fig. 1:**
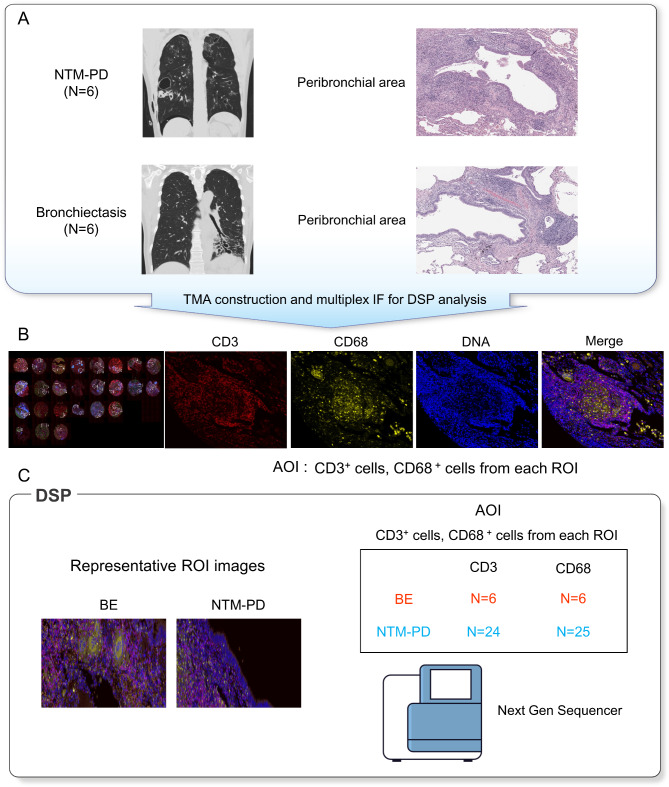
Overview of digital spatial profiling (DSP) workflow. **(a)** Schematic of study participants. Lung specimens were obtained from six patients undergoing surgical resection for nontuberculous mycobacterial pulmonary disease (NTM-PD) and six patients with bronchiectasis. Lesion samples from the peribronchial areas were collected and used to create tissue microarray (TMA) slides for DSP analysis. **(b)** Representative images of a multi-label immunofluorescence scan of a TMA sample and representative immunofluorescence staining features of each marker. CD3 (red), CD68 (yellow), DNA (DAPI), and a merged staining image are shown. **(c)** Representative images of peribronchial areas in patients with NTM-PD and bronchiectasis. RNA sequencing analyses were performed using CD3- and CD68-positive cells from each region-of-interest

TMAs were constructed from formalin-fixed paraffin-embedded tissue blocks. We obtained 2-mm-diameter cores from the peribronchial areas, which were assessed by two lung pathologists with more than 10 years of experience. A total of 25 tissue cores from 12 patients (six with NTM-PD and six with bronchiectasis) were arrayed and used for subsequent DSP analysis. Additionally, 45 cores from 45 patients (30 with NTM-PD and 15 with bronchiectasis) were prepared for IHC analysis (Fig. 1A).

### RNA sequencing and data analysis

TMA sections were stained simultaneously with fluorescently labeled antibodies specific for the T cell marker CD3, macrophage marker CD68, and nuclear stain DNASyto13 for DSP. For each peribronchial core, more than one region-of-interest (ROI) of up to 660 × 780 µm^2^ was selected. Each ROI was separately assessed for the proportion of CD3^+^ T cells and CD68^+^ macrophages as an area-of-interest (AOI) (Fig. 1B). Following probe hybridization, ultraviolet-induced cleavage, and barcode collection, gene expression at the whole-transcriptome level was quantified by PCR amplification and Illumina sequencing (NanoString Technologies, Seattle, WA, USA; Fig. 1C).

For the transcriptomic analysis, the 75th percentile (Q3) of the gene counts of each probe in each AOI was calculated and normalized to the geometric mean of the 75th percentile across all AOIs. Normalized data were used for analysis. Gene set enrichment analysis (GSEA) was performed using GSEA software (version 4.3.2) with hallmark gene sets and the Gene Ontology Biological Process.

### IHC

IHC for CD3 (F7.2.38, Dako, 1:300), CD8 (SP16, Thermo Fisher Scientific, 1:100), CD68 (PG-M1, Dako, 1:50), CD103 (EPR4166, Abcam, 1:800), PD-1 (MRQ-28, Cell marque, 1:100), Foxp3 (236 A/E7, Abcam, 1:50), TIM-3 (#45208, Cell Signaling Technology, 1:200), CD11c (5D11, Leica Biosystems, 1:200), CD163 (MRQ-26, Cell Marque, 1:200), granzyme β (262 A-15, Cell marque, 1:50), and NF-κB p65 (#8242, Cell Signaling Technology, 1:1000) was performed using a Benchmark XT autostainer (Ventana Medical Systems).

Whole slide images were obtained using virtual microscopic scanning of the IHC slides with an Aperio ScanScope (Aperio Technologies, Vista, CA, USA). CD3-, CD4-, CD8-, CD103-, PD-1-, and TIM-3-positive lymphoid cells and CD68- and CD163-positive cells were automatically counted using QuPath software [12]. The cell counts were converted to cell density (cell count/mm^2^).

### Statistical analysis

Data visualization, including heatmaps, principal component analysis (PCA) plots, and immune cell fractions, was performed using the GeoScript Hub provided on the NanoString platform and GraphPad Prism (version 8.0; GraphPad Software, San Diego, CA, USA). The cutoff value for differentially expressed genes (DEGs) was set at log_2_-FC > 1.5 and adjusted *p* < 0.05. All statistical analyses were performed using R software (version 4.3.2; R Foundation for Statistical Computing, Vienna, Austria). A two-sided *p* < 0.05 was considered statistically significant.

## Results

### NTM-PD reveals distinct gene expression patterns and pathway activation according to cell type

A total of 25 tissue cores obtained from 12 patients were arrayed for DSP (Fig. 1A) and stained with fluorescently labeled antibodies (Fig. 1B). Gene expression according to the area and cell type was quantified using PCR amplification and Illumina sequencing (Fig. 1C).

**Fig. 2:**
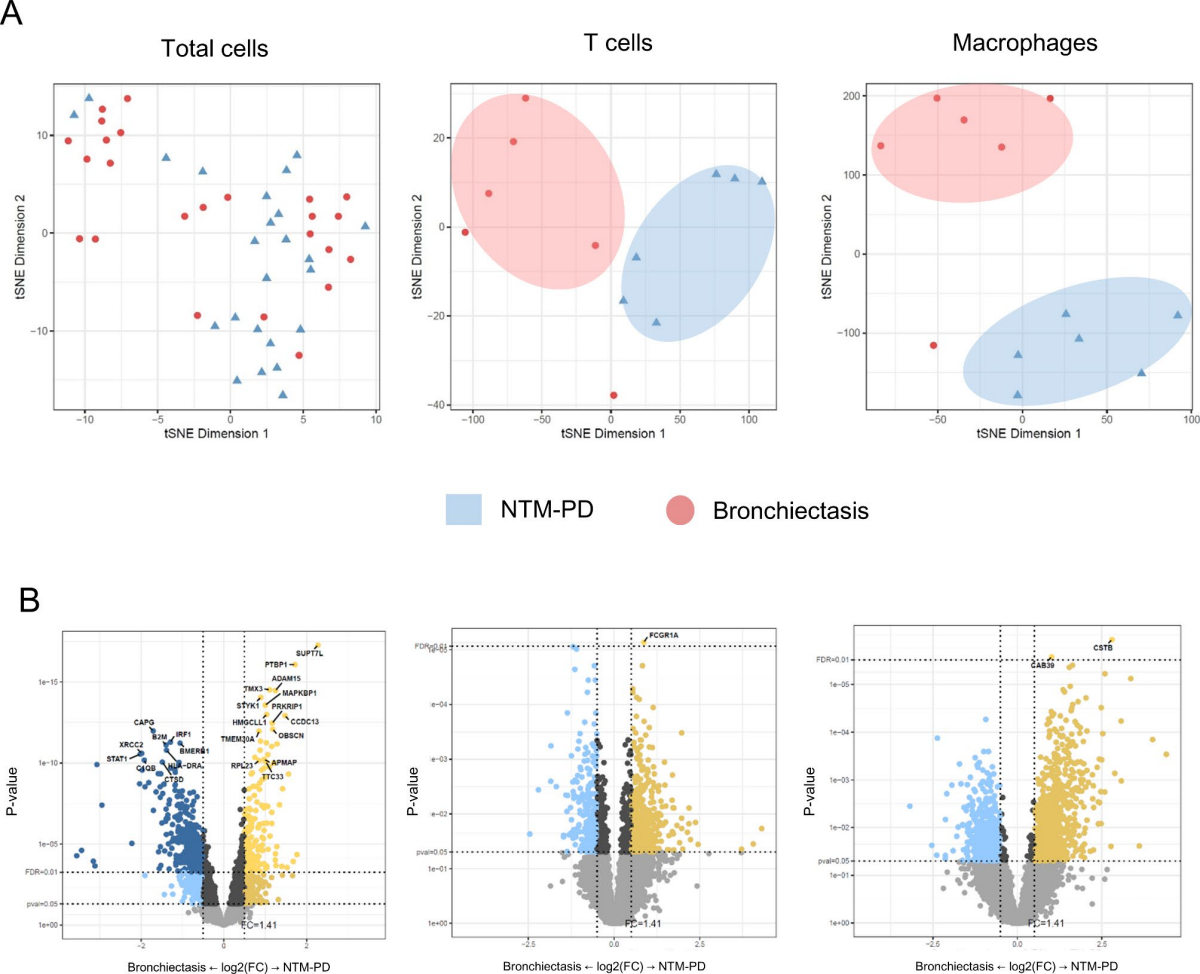
Identification of differentially expressed genes. **(a)** Principal component analysis plots of RNA sequencing data illustrating RNA expression patterns of total cells, T cells, and macrophages in lung tissues from patients with NTM-PD or bronchiectasis. **(b)** Volcano plots of gene expression changes in total cells, T cells, and macrophages between NTM-PD and bronchiectasis. Yellow dots indicate genes upregulated in NTM-PD, while dark blue dots indicate genes upregulated in bronchiectasis samples (*p* < 0.05)

PCA plots revealed similar mRNA expression patterns in NTM-PD and bronchiectasis (Fig. 2A). However, at the cell-type level, such as T cells and macrophages, the mRNA expression profiles of NTM-PD and bronchiectasis exhibited clear spatial differences. We identified 1,289 DEGs between NTM-PD and bronchiectasis (Fig. 2B). A list of the top 50 distinct genes between NTM-PD and bronchiectasis by cell type is provided in the Supplementary file.

**Fig. 3:**
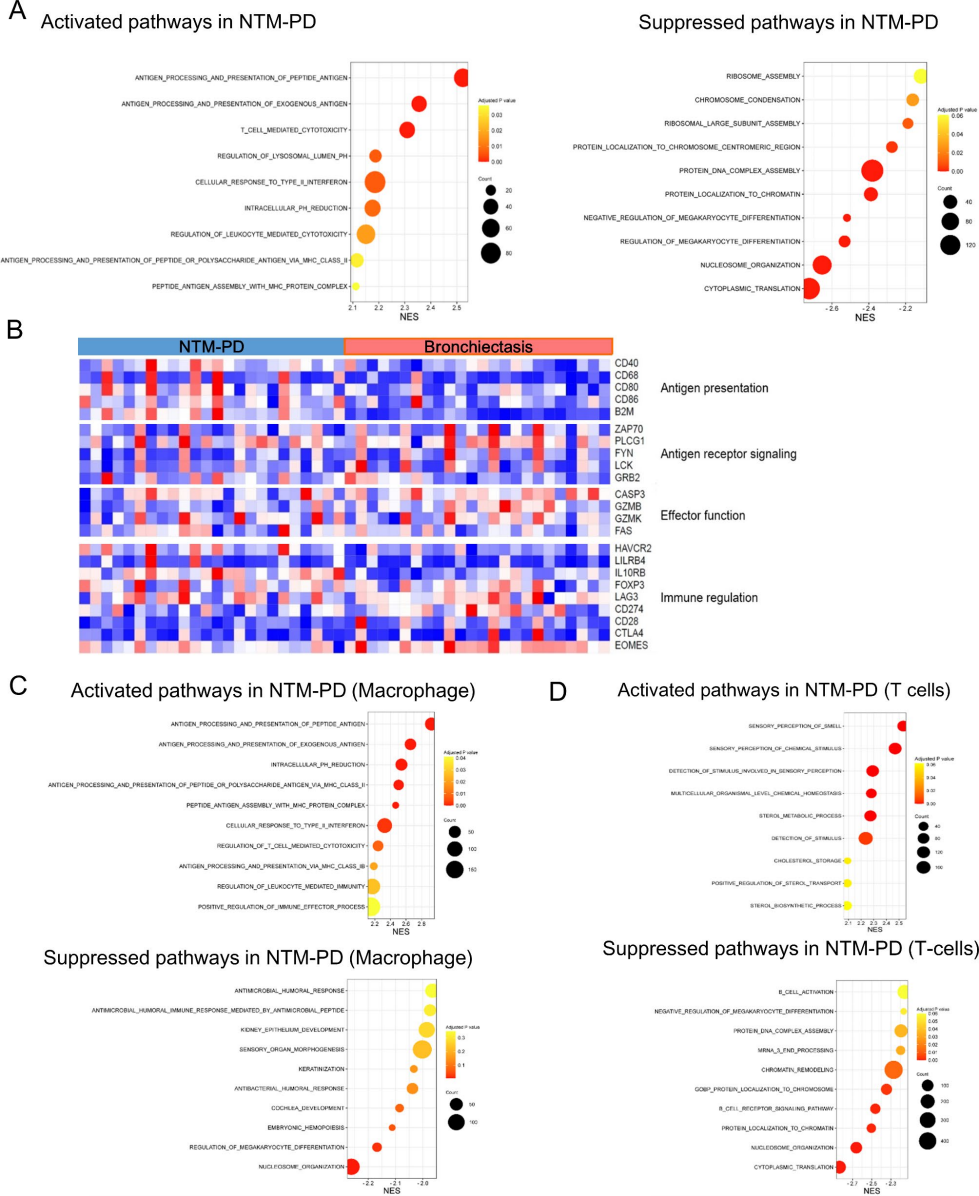
Pathways enriched in NTM-PD and bronchiectasis. **(a–d)** Data from total immune cells between NTM-PD and bronchiectasis. **(a)** Dot plot of gene set enrichment analysis (GSEA) displaying activation and inactivation of significantly enriched signaling pathways in total cells from NTM-PD compared with bronchiectasis. **(b)** Expression pattern of genes involving antigen presentation, antigen receptor signaling, effector function, and immune regulation in total cells. (c–d) Dot plots of GSEA displaying activation and inactivation of significantly enriched signaling pathways in macrophages **(c)** and T cells **(d)** from NTM-PD compared with bronchiectasis

In the GSEA of the total cells, pathways related to antigen processing and presentation, T cell-mediated cytotoxicity, and cellular response to type II interferons were activated in NTM-PD, whereas cytoplasmic translation, nucleosome organization, and protein localization to chromatin were downregulated (Fig. 3A). Heatmap clustering of genes associated with immune responses to microbial infection revealed a tendency towards the increased expression of genes related to antigen presentation (Fig. 3B).

Pathways associated with antigen processing and presentation were activated in macrophages, similar to the observations in total cells (Fig. 3C), whereas the same pathways were not activated in T cells. Reception-related pathways were increased in NTM-PD, whereas pathways related to B cell receptor signaling, cytoplasmic translation, and nucleosome organization were decreased (Fig. 3D).

### M1-phenotype macrophages dominate in NTM-PD

**Fig. 4:**
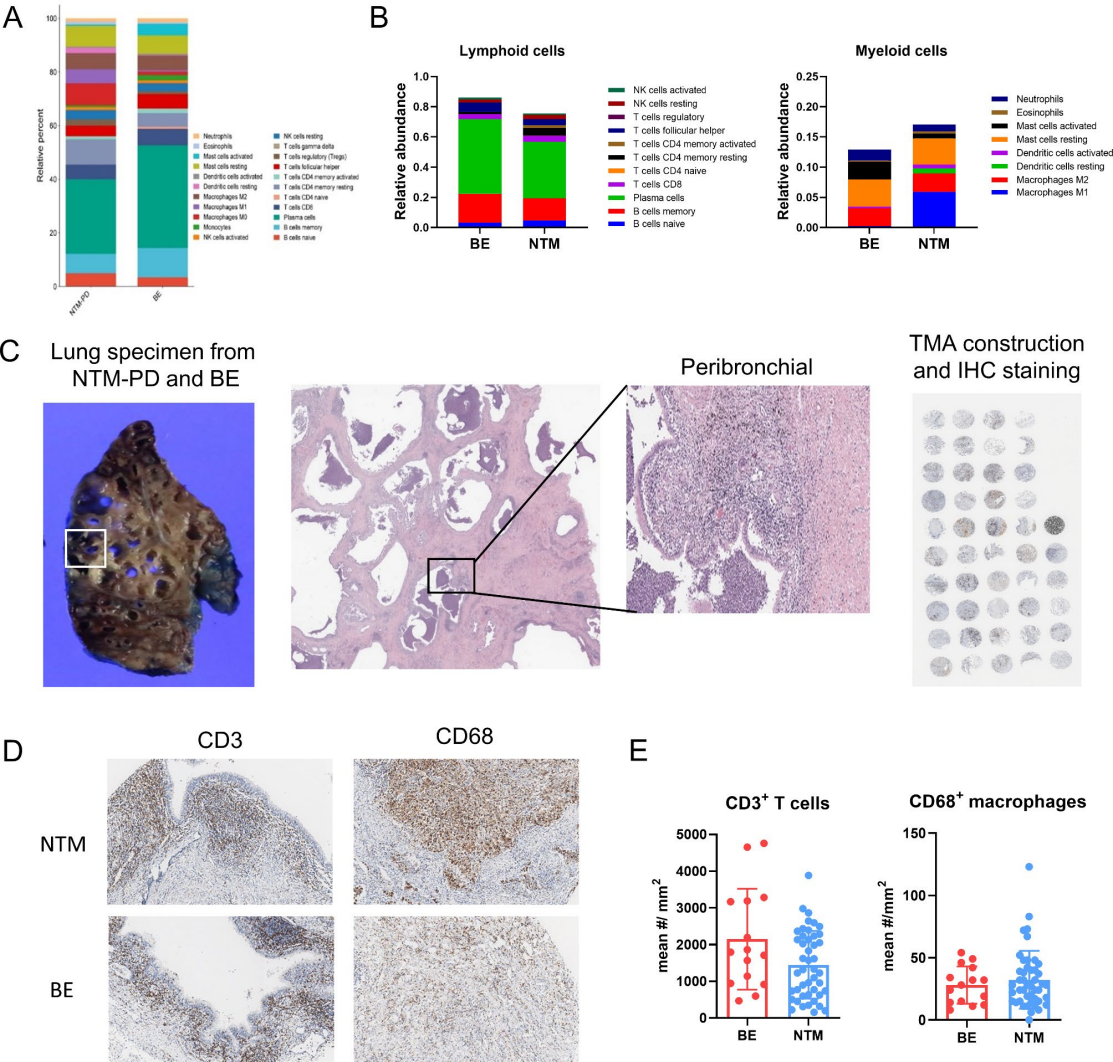
Distribution of immune cells according to disease and validation. **(a)** Stacked bar charts comparing proportions of immune cell subsets in peribronchial area between NTM-PD and bronchiectasis. **(b)** Comparison of the relative abundance of lymphoid and myeloid cells between groups. **(c)** Schematic overview of TMA construction for validation cohort. TMAs were constructed using samples from the peribronchial areas of patients with NTM-PD and bronchiectasis, respectively, and immunohistochemical staining for immune cell markers was performed. **(d)** Representative images of immunohistochemical staining for CD3 (T-lymphocyte marker) and CD68 (macrophage marker) in the two groups. **(e)** Bar graphs showing mean number of CD3^+^ lymphocytes and CD68^+^ macrophages between the groups

Immune cell populations in the ROIs are shown in Fig. 4A. Although lymphoid cells were less prevalent, myeloid cells were more prevalent in the NTM-PD group than in the bronchiectasis group (Fig. 4B). To validate these findings, we performed IHC using TMAs obtained from another cohort (Fig. 4C). CD3^+^ T cells (representative lymphoid cells) and CD68^+^ macrophages (representative myeloid cells in the lungs) were quantified through immunohistochemical staining for CD3 and CD68 (Fig. 4D). The number of CD3^+^ T cells was lower in NTM-PD than in bronchiectasis, whereas the number of CD68^+^ macrophages was higher (Fig. 4E).

**Fig. 5:**
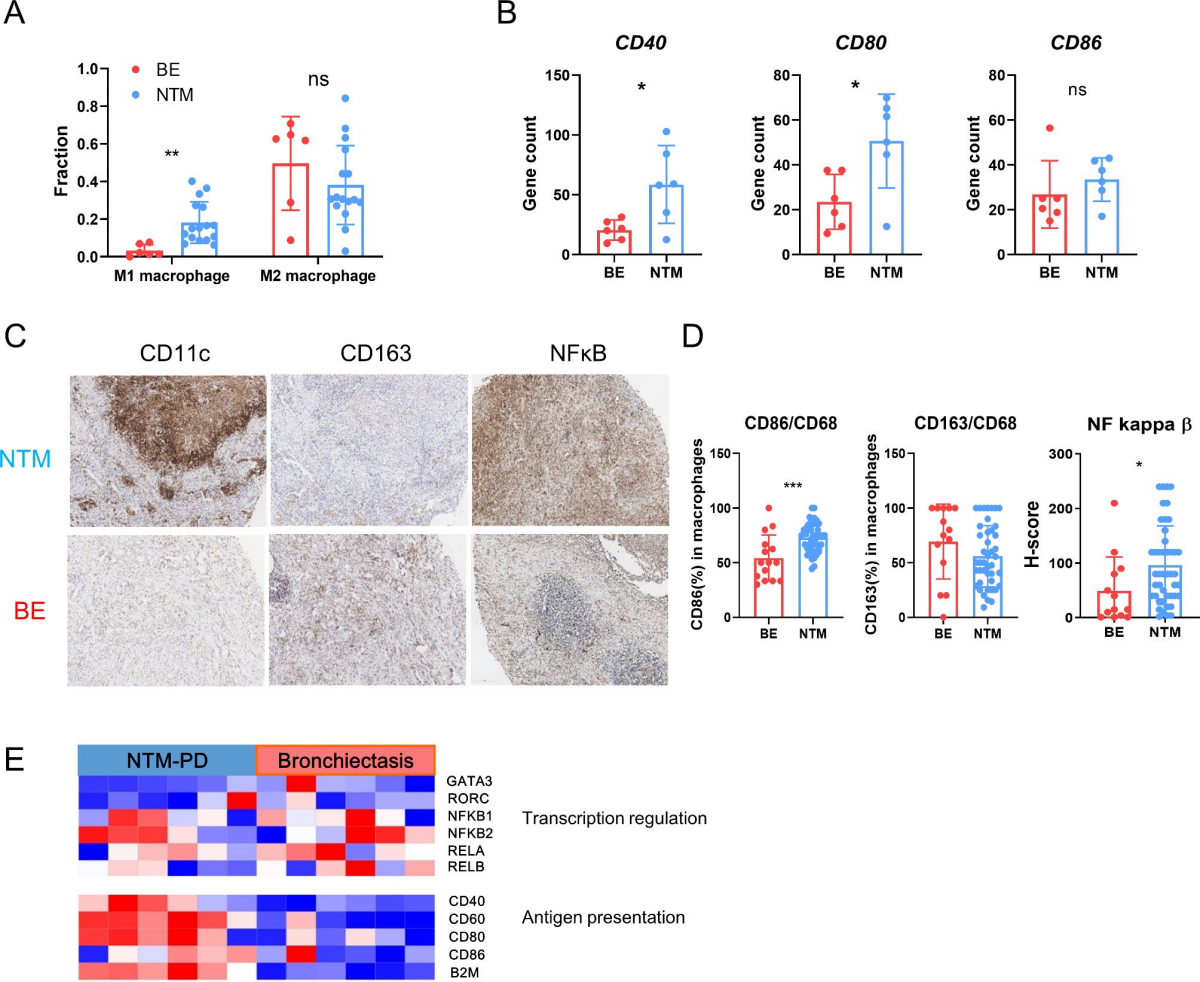
Macrophages exhibit dominant M1 phenotype in NTM-PD. **(a)** Bar graph showing proportions of M1 and M2 macrophages between NTM-PD and bronchiectasis. **(b)** Boxplots comparing levels of *CD80*,* CD40*, and *CD86* between the groups. **(c)** Representative images of immunohistochemical staining for CD68, CD11c, CD163, and NF-κB in the two groups. **(d) ** Plots showing proportions of M1 macrophage (CD11c^+^ per CD68^+^ cells), M2 macrophage (CD163^+^ per CD68^+^ cells), and H-score of NF-κB between the groups. **(e)** Heatmap of genes involving transcription regulation and antigen presentation in macrophages from the two groups

Among the macrophages in the NTM-PD group, the increase in the number of M1 macrophages was most pronounced (Fig. 5A). The macrophage expression of genes associated with the M1 phenotype (*CD40* and *CD80*) was significantly elevated in NTM-PD (Fig. 5B). This increase in M1 macrophages was corroborated by IHC, which showed elevated CD11c (representative marker of M1 macrophages) and decreased CD163 (representative marker of M2 macrophages) expression in the NTM-PD group (Fig. 5C and D). These macrophages exhibited increased activity, as evidenced by the elevated expression of genes associated with antigen presentation (Fig. 5E), consistent with the increased expression of NF-κB in NTM-PD (Fig. 5C and D).

### Macrophage activation does not lead to T cell activation in NTM-PD

**Fig. 6:**
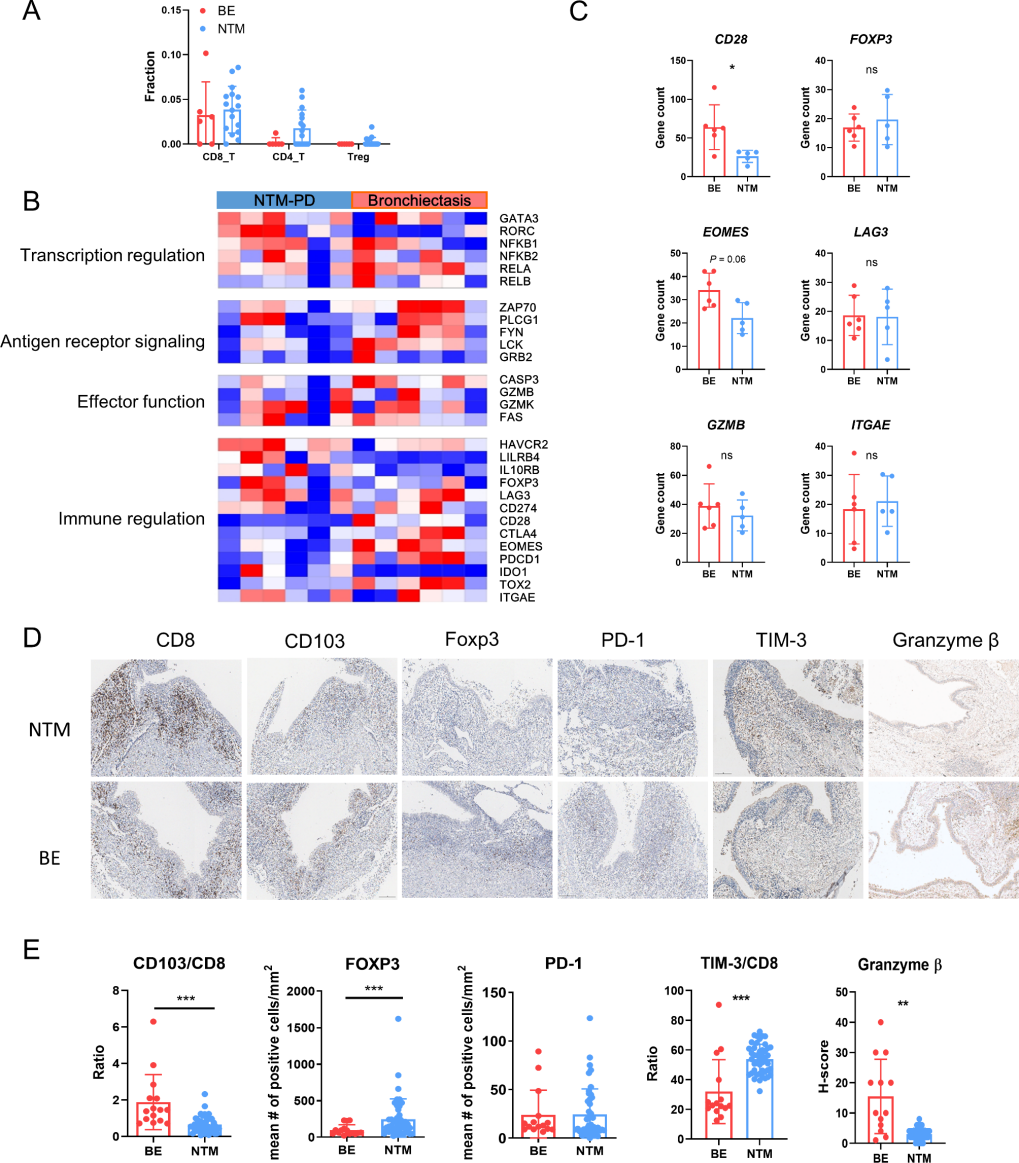
Absence of T cell activation following macrophage activation in NTM-PD. **(a)** Comparison of CD8, CD4, and regulatory T cell proportions in RNA sequencing data between NTM-PD and bronchiectasis. **(b)** Heatmap of genes involving transcription regulation, antigen presentation, effector function, and immune regulation in T cells from the two groups. **(c)** Expression of *CD28*,* EOMES*,* GZMB*,* FOXP3*,* LAG3*, and *ITGAE* determined using RNA sequencing in the two groups. **(d)** Representative images of immunohistochemical staining for CD8, CD103, Foxp3, PD-1, TIM-3, and granzyme β in the two groups. **(e)** Plots showing the ratio of CD103^+^ to CD8^+^ T lymphocytes, number of Foxp3^+^ or PD-1^+^ T lymphocytes, ratio of TIM-3^+^ to CD8 T^+^ lymphocytes, and H-score for granzyme β between the groups

CD8^+^, CD4^+^, and regulatory T cells counts were higher in NTM-PD than in bronchiectasis (Fig. 6A). However, contrary to our expectations, the effector functions of these T cells were not enhanced (Fig. 6B): The expression of *CD28*, which is crucial for T cell co-stimulation, was reduced (Fig. 6C). These findings were further supported by the IHC analysis (Fig. 6D). Although PD-1-positive lymphocytes showed no difference between NTM-PD and bronchiectasis, the levels of T cells expressing Foxp3 or TIM-3 increased in NTM-PD, whereas the expression of granzyme B decreased. These results suggest that, although M1 macrophages are stimulated in NTM-PD, this does not translate into enhanced T cell functioning.

## Discussion

In this study, we analyzed the distribution and function of immune cells in the NTM-PD microenvironment based on their RNA and protein expression. Specifically, we delineated the distinct immunologic signatures between NTM-PD and bronchiectasis at the cellular level, focusing on T cells and macrophages. Despite the notable activation of M1 macrophages, no T cell activation was observed, suggesting that the pathogenesis of NTM-PD is unique relative to bronchiectasis.

NTM-PD is closely associated with bronchiectasis. Persistent NTM infection can lead to the development of bronchiectasis, and the presence of bronchiectasis can increase susceptibility to NTM-PD [8, 13]. Additionally, NTM-PD and bronchiectasis exhibit nearly identical metabolomic profiles [14]. Therefore, the roles of immune cells are expected to be similar in NTM-PD and bronchiectasis. However, our study revealed distinct immunologic features in NTM-PD. t-Distributed stochastic neighbor embedding plots showed clear differences in the immune responses between NTM-PD and bronchiectasis at the T cell and macrophage levels. This suggests that, despite their phenotypic similarities, NTM-PD exhibits distinct immunologic features.

We observed the upregulation of pathways related to antigen presentation and processing in NTM-PD, particularly in macrophages, compared to that in bronchiectasis. This finding is plausible, given the role of macrophages in mycobacterial defense mechanisms. Alveolar macrophages are the first line of defense against mycobacterial pathogens in the lower respiratory tract [15]. Macrophages are activated upon recognition of pathogen-associated molecular patterns such as lipomannan, trehalose dimycolate, or PI-mannoside presented by NTM [16]. This activation leads to the synthesis of nitrogen and oxygen radicals, formation of phagolysosomes, and induction of autophagy [16, 17]. Moreover, as antigen-presenting cells, macrophages play a crucial role in the differentiation of naïve T cells into Th1 cells [18].

NTM infection is known to induce M1 macrophage polarization, characterized by the increased production of nitric oxide, IFN-γ, IL-1β, and IL-6 [19, 20]. This polarization is facilitated by the activation of NF-κB, a major transcription factor that regulates pro-inflammatory mediator expression during mycobacterial infection [20, 21]. In our study, we observed an increase in the number and function of macrophages in patients with NTM-PD. Notably, these macrophages were polarized towards the M1 rather than the M2 phenotype. We confirmed the activation of NF-κB pathways in macrophages through IHC. The predominance of the M1 phenotype is expected to promote the eradication of NTM [20].

Despite our findings of M1 macrophage activation in NTM-PD, its complete pathogenesis and the causes of refractory treatment responses remain unclear. In our study, the most activated pathway in T cells was related to olfactory receptor stimulation. This stimulation is known to induce the chemotaxis of T cells expressing the olfactory receptor, even in extra-nasal organs including the lung [22, 23]. At the same time, however, the evidence of immune regulation by T cells was also observed in our study. Specifically, the expression of T cell co-stimulatory molecules, such as CD28, decreased, while the abundance of regulatory T cells, indicated by the presence of Foxp3 [24], increased. Moreover, the expression of TIM-3, which increases the severity of mycobacterial infections when expressed on CD8^+^ T cells [25], was elevated in NTM-PD. The absence of T cell activation coupled with macrophage activation in NTM-PD could explain the treatment refractoriness observed in the study subjects.

Our study had some limitations. First, the number of subjects for whom DSP analysis was performed was small. However, to mitigate the limitations of small sample size, we validated our findings using IHC in a larger cohort. Second, the patients with NTM-PD were limited to those who underwent surgical resection because of tissue availability, potentially limiting the generalizability of our findings. Nonetheless, this study has several strengths. To the best of our knowledge, this is the first study to investigate the immunologic profiles of NTM-PD using DSP, which effectively elucidated the cell-level immunologic signatures within target lesions. Specifically, by comparing it with bronchiectasis, we minimized noise from preexisting structural lung disease and identified the immunologic features provoked by NTM itself.

## Conclusions

In conclusion, NTM-PD exhibits distinct immunologic signatures characterized by the activation of macrophages couple with little T cell activation. Our findings provide new insights into the pathogenesis of NTM-PD.

### Electronic supplementary material

Below is the link to the electronic supplementary material.

Supplementary Material 1

## Data Availability

The datasets used and/or analysed during the current study are available in the Gene Expression Omnibus (accession number: GSE273714).

## Abbreviations

AOI: area-of-interest
DSP: digital spatial profiling
IHC: immunohistochemistry
NTM-PD: nontuberculous mycobacterial pulmonary disease
ROI: region-of-interest
TMA: tissue microarray
